# Quantitative proteomics and phosphoproteomics reveal insights into mechanisms of *ocnus* function in *Drosophila* testis development

**DOI:** 10.1186/s12864-023-09386-2

**Published:** 2023-05-26

**Authors:** Ya Zheng, Bin Mao, Qian Wang, Xin Duan, Meng-Yan Chen, Wei Shen, Chao Li, Yu-Feng Wang

**Affiliations:** School of Life Sciences, Hubei Key Laboratory of Genetic Regulation and Integrative Biology, Central China Normal University, Wuhan, 430079 P. R. China

**Keywords:** *Ocnus*, *Drosophila melanogaster*, Proteomics, Phosphoproteomics, Testis development

## Abstract

**Background:**

Testis is the only organ supporting sperm production and with the largest number of proteins and tissue-specific proteins in animals. In our previous studies, we have found that knockdown of *ocnus* (*ocn*), a testis-specific gene, resulted in much smaller testis with no germ cells in *Drosophila melanogaster*. However, the molecular consequences of *ocn* knockdown in fly testes are unknown.

**Results:**

In this study, through iTRAQ quantitative proteomics sequencing, 606 proteins were identified from fly abdomens as having a significant and at least a 1.5-fold change in expression after *ocn* knockdown in fly testes, of which 85 were up-regulated and 521 were down-regulated. Among the differential expressed proteins (DEPs), apart from those proteins involved in spermatogenesis, the others extensively affected biological processes of generation of precursor metabolites and energy, metabolic process, and mitochondrial transport. Protein-protein interaction (PPI) analyses of DEPs showed that several kinases and/or phosphatases interacted with Ocn. Re-analyses of the transcriptome revealed 150 differential expressed genes (DEGs) appeared in the DEPs, and their changing trends in expressions after *ocn* knockdown were consistent. Many common down-regulated DEGs and DEPs were testis-specific or highly expressed in the testis of *D. melanogaster.* Quantitative RT-PCR (qRT-PCR) confirmed 12 genes appeared in both DEGs and DEPs were significantly down-regulated after *ocn* knockdown in fly testes. Furthermore, 153 differentially expressed phosphoproteins (DEPPs), including 72 up-regulated and 94 down-regulated phosphorylated proteins were also identified (13 phosphoproteins appeared in both up- and down-regulated groups due to having multiple phosphorylation sites). In addition to those DEPPs associated with spermatogenesis, the other DEPPs were enriched in actin filament-based process, protein folding, and mesoderm development. Some DEPs and DEPPs were involved in Notch, JAK/STAT, and cell death pathways.

**Conclusions:**

Given the drastic effect of the *ocn* knockdown on tissue development and testis cells composition, the differences in protein abundance in the *ocn* knockdown flies might not necessarily be the direct result of differential gene regulation due to the inactivation of *ocn*. Nevertheless, our results suggest that the expression of *ocn* is essential for *Drosophila* testis development and that its down-regulation disturbs key signaling pathways related to cell survival and differentiation. These DEPs and DEPPs identified may provide significant candidate set for future studies on the mechanism of male reproduction of animals, including humans.

**Supplementary Information:**

The online version contains supplementary material available at 10.1186/s12864-023-09386-2.

## Background

With the in-depth study of the reproductive development mechanism in *Drosophila*, scientists have discovered that the testis development is more complicated [1]. In view of the decline in male fertility in recent decades, and age-related male infertility in developed countries, there is an urgent need for further improvement in the understanding of the mechanisms of male reproduction [2]. Spermatogenesis is a highly conserved process in many animal taxa, from *Drosophila* in insects to mice in mammals, including several common steps: differentiation of germline stem cells to spermatogonia, growth of spermatogonia to spermatocytes, meiotic divisions to generate haploid spermatids, and spermiogenesis (morphogenesis) [3–5]. *Drosophila* is an excellent model for deciphering the mechanisms of spermatogenesis as the morphology of germ cells is visibly trackable at different stages and over 65% of the updated *D. melanogaster* sperm proteome has mammalian orthologs in both mouse and human [6, 7]. Proteomics will play an important role in promoting these advances in the study of human fertility [8]. The comparative analysis of omics between control testis and defective testis of *Drosophila* may provide a targeted set of candidates for further studies on their functions in male reproduction of animals [9].

In the testis of *D. melanogaster*, germline cells continuously develop and differentiate depending on intimate contact with somatic cells, including hub cells and cyst cells. The interactions between germline and soma are very important for sperm generation and function [10]. The signals, such as JAK/STAT, BMP, and Hedgehog, from the hub at the apical tip of the testis support two neighboring stem cell populations, Germ stem cells (GSCs) and Cyst stem cells (CySCs) [10, 11]. Two CySCs encapsulate one GSC and exchange signals with it. GSCs and CySCs divide in an asymmetrical way to produce two types of daughter cells. The ones that directly contact with the hub receive these signals and maintain the stem cell state, while the ones displaced from the hub (gonialblasts and cyst cells, respectively) form developmental units called cysts. Two cyst cells enclose one gonialblast, forming a cyst. Then the gonialblast undergoes four rounds of mitotic divisions to produce 16 spermatogonia, which then develop to spermatocytes. After meiosis and dramatic morphological changes, 64 elongated spermatids are formed in one cyst [10]. During all these developmental stages, the two cyst cells always enclose, grow, and co-differentiate with the germline cells, regulating their survival, division, and differentiation [10, 11]. Defects in these somatic cells in testes result in the failure of the production of functional sperms [10–12].

*ocnus* (*ocn*), along with two related genes *janusA* (*janA*) and *janusB* (*janB*), are arranged in tandem in a region of less than 2.5 kb on chromosome arm 3R [13, 14]. The phylogenetic analysis based on protein-encoding sequences of JanA, JanB, and Ocn, as well as their physical proximity, suggested a duplication pattern of *janA* → *janA janB*→ *janA janB ocn*. *janA* resembles the most ancestral sequence, and after two separate duplication events *ocn* was produced. Contrary to *janA*, which shows a tissue and developmental broad pattern of expression in both males and females, *janB* and *ocn* exhibit testis-specific expression [13, 14]. These indicate that following the duplication events the new produced genes increase specificity of functions. The mammalian ortholog of Ocn protein is phosphohistidine phosphatase 1 (PHPT1), which is involved in peptidyl-histidine dephosphorylation. PHPT1 also regulates ATP-citrate lyase (ACLY) activity or cell viability in neuroblastomas [15]. Moreover, the histidine phosphatase homologue in *Caenorhabditis elegans* is only expressed in neurons, indicating that histidine dephosphorylation has a unique role in neuronal function [16]. However, the role of histidine phosphatases in *Drosophila* development, especially in testis development, is not understood.

We have previously reported that knockdown of *ocn* in the testis of *D. melanogaster* led to much smaller testes [17]. Immunostaining analysis showed that these small testes contained no germ cells but extended hub somatic cells, which are normally restricted to the apical tip of fly testes. By RNA-sequencing, we identified many differentially expressed genes after *ocn* knockdown in fly testes [17]. To further investigate the molecular mechanisms by which *ocn* affects fly testis development, we dissected the fly abdomens (as the *ocn* knockdown fly testis was too small to be dissected and obtain enough proteins) and used a global analysis of iTRAQ-based quantitative proteome coupled with phosphopeptide-enrichment strategies to reveal the differentially expressed proteins (DEPs) and differentially expressed phosphorylated proteins (DEPPs) after *ocn* knockdown. Considering the severe effect of the *ocn* knockdown on tissue development and testis cells composition, the differences in protein abundance in the *ocn* knockdown flies might not necessarily be the direct result of differential gene regulation due to the depletion of *ocn*. Nevertheless, our results suggest that the expression of *ocn* is important for *Drosophila* testis development and that its down-regulation impairs key signaling pathways related to cell survival and differentiation.

## Results

### Overexpression of ***ocn*** can partially rescue male infertility caused by ***ocn*** knockdown

**Fig. 1:**
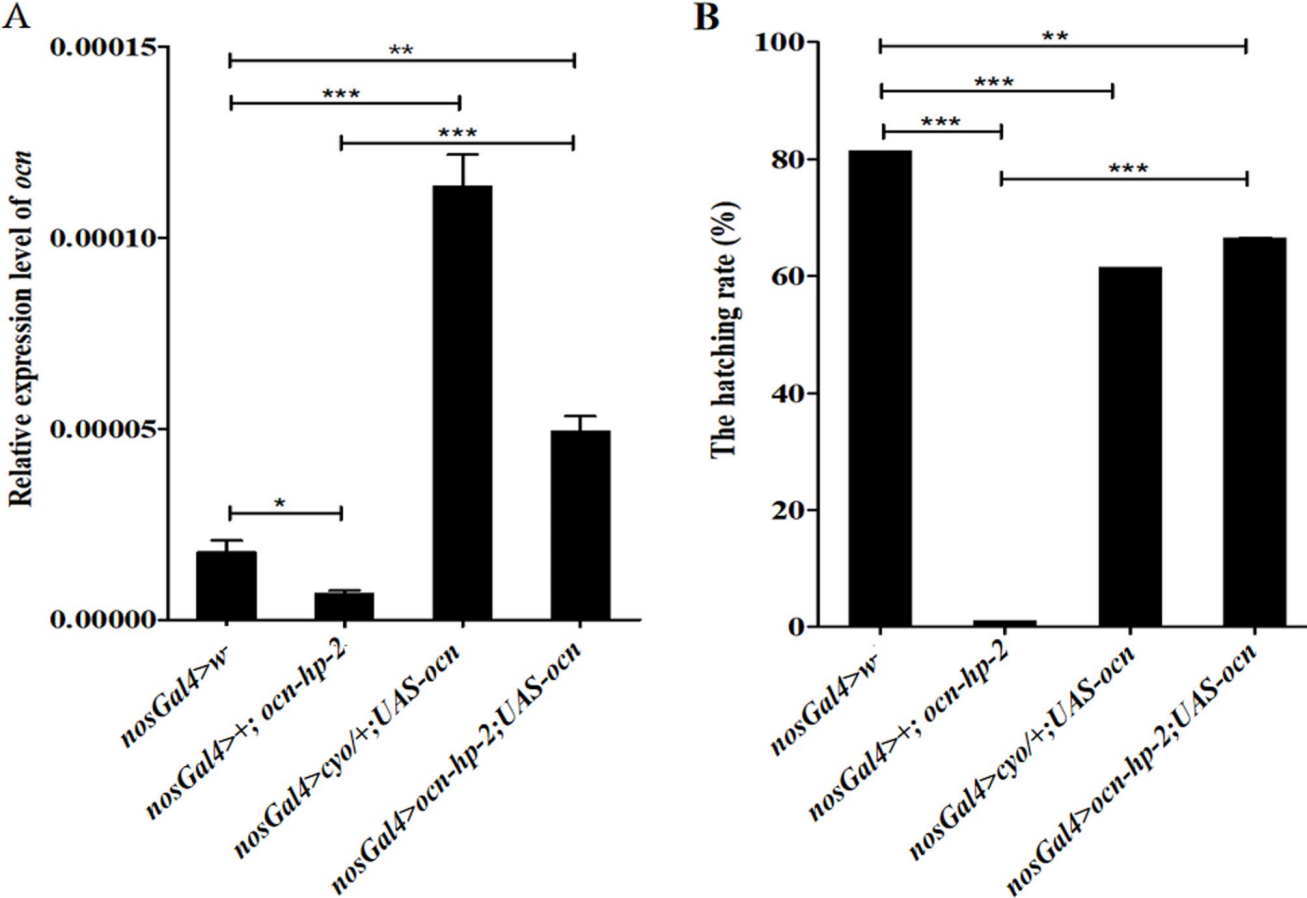
Overexpression of *ocn* can partially rescue male infertility caused by *ocn* knockdown. (A) qRT-PCR analyses of the expression levels of *ocn* in testes from *nosGal4 > w*^*−*^ (control), *nosGal4 > ocn-hp-2* (knockdown), *nosGal4 > cyo/+;UAS-ocn *(overexpression), and *nosGal4 > ocn-hp-2;UAS-ocn* (rescue). (B) Hatching rates of eggs derived from the crosses with the corresponding males. Error bars are standard errors. * *P* < 0.05; ** *P* < 0.01; *** *P* < 0.001 (Student’s *t*-test)

We have previously reported that knockdown of *ocn* resulted in male sterility. To avoid the possibility that the phenotypes caused by *ocn* knockdown arise from off-target effects, we first experimented with a second *ocn* RNAi line, which interferes a much longer part of *ocn* coding sequence (see “Methods”), on its role in male fertility. In accord with our previous result, the expression level of *ocn* was significantly decreased in the testis of *nosGal4*>*+*; *ocn-hp-2* flies compared with the control (*nosGal4 > w*^*−*^) (*P* < 0.05) (Fig. 1A). Correspondingly, the depletion of *ocn* in fly testes also resulted in almost sterility of male *D. melanogaster* (Fig. 1B), the hatching rate of eggs derived from the crosses with *ocn* knockdown males (*nosGal4*>*+*; *ocn-hp-2*) was only 1.07 ± 0.003%. Then we used the overexpressing line (*UAS-ocn*) to rescue the *ocn* knockdown phenotype, and found that male fertility was significantly restored. As shown in Fig. 1B, the hatching rate of eggs from the groups crossed with *nosGal4* > *ocn-hp-2; UAS-ocn* males was significantly higher (66.47 ± 0.02%,) than that in *ocn* knockdown groups, and the *ocn* expression level was also significantly increased in the testis of *nosGal4* > *ocn-hp-2; UAS-ocn* males when compared with that in the *ocn* knockdown groups (Fig. 1A), although the egg hatch rate was still lower than that in control group, which could be due to that too high expression level of *ocn* resulting from the *UAS-ocn* transgene is also toxic to male fertility (Fig. 1). This result indicates that *ocn* is indeed essential for male fertility in *D. melanogaster*.

### Protein identification and quantification of the ***ocn*** knockdown and control male fly abdomens

**Fig. 2:**
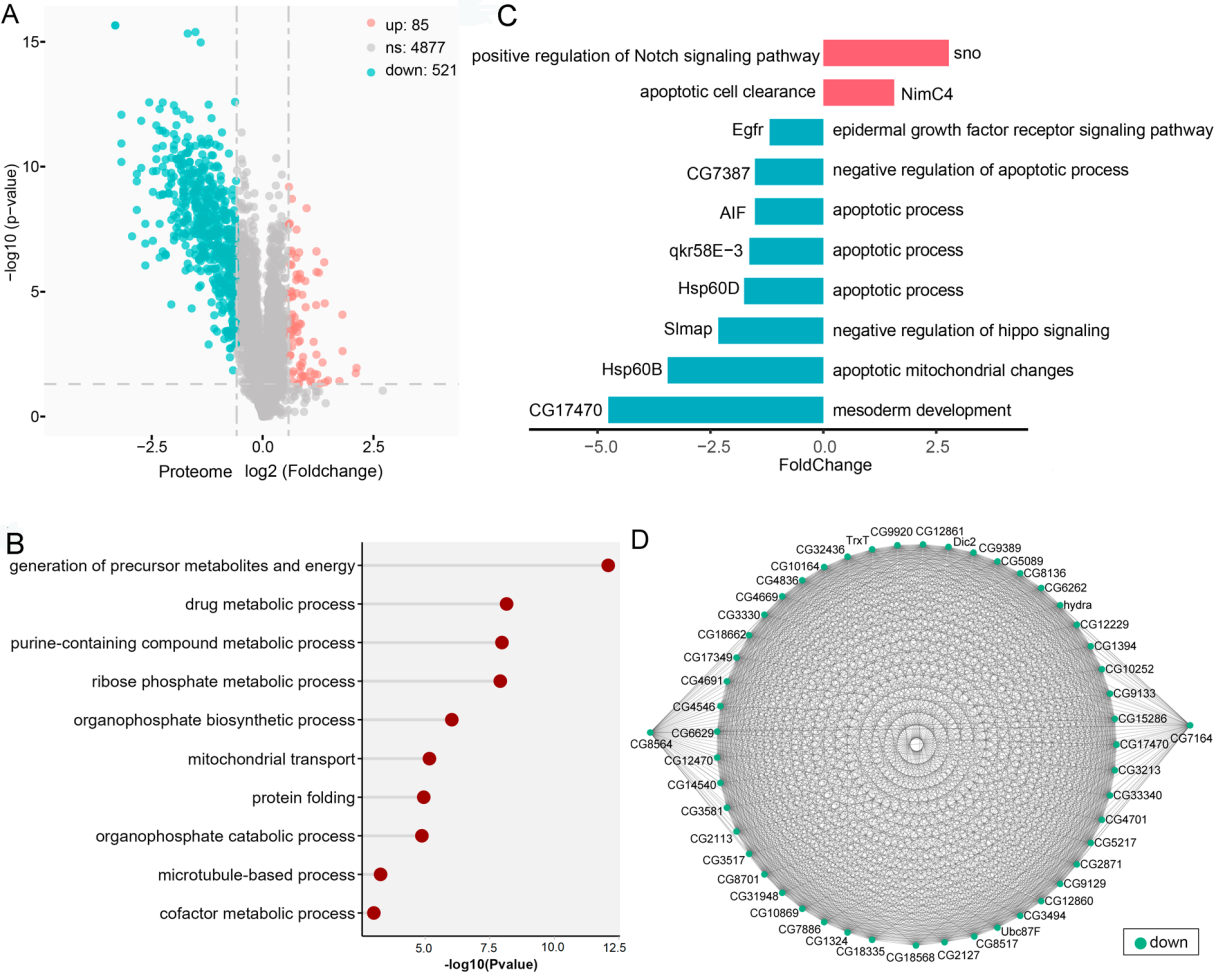
Analysis of differentially expressed proteins (DEPs) between the *ocn* knockdown (nosGal4 > *ocn-*hp) and control (nosGal4 > *w*^*–*^) male fly abdomens. **(A)** Volcano plot of the quantified proteins in all biological replicates (the significant down-regulated and up-regulated proteins shown in the cyan and red plots, respectively). **(B)** GO biological process enrichment analysis of the DEPEs (DEPs that excluded those involved in spermatogenesis). **(C)** Log2 (fold change) of the expression levels of DEPEs involved in key pathways involved in testis development. **(D)** Protein-protein interaction analysis of DEPEs.

To explore the mechanisms by which *ocn* knockdown led to defects in testis development in *D. melanogaster*, we performed iTRAQ labeling of abdominal proteins from *ocn* knockdown (nosGal4 > *ocn-hp*) and control (nosGal4 > *w*^*–*^) male flies, respectively, and quantified 29,542 peptides corresponding to 5,483 proteins with FDR (False positive rate) < 1.0%. Among these proteins, 606 were identified as DEPs based on a cutoff of 1.5-fold change and *P* < 0.05. Of which, 85 were up-regulated and 521 were down-regulated after *ocn* knockdown (Fig. 2A, Additional file 1).

### Functional classification of DEPs

To analyze the biological functions of the DEPs, we performed GO biological processes (GOBP) enrichment, and found that proteins associated with spermatogenesis, such as cilium movement, microtubule-based processes, and spermatid differentiation were significantly enriched. This is consistent with the defects showing in male germ cell development after *ocn* knockdown in fly testes [17]. We then excluded spermatogenesis related proteins in DEPs and re-analyzed the left DEPs (DEPEs), GOBP analyses showed that most of DEPEs were enriched in generation of precursor metabolites and energy, metabolic process, mitochondrial transport, and protein folding (Fig. 2B). Considering the phenotype of much smaller size of the testis with no germ cells but extended somatic hub cells caused by *ocn* knockdown, we extracted DEPEs involved in germ cell differentiation and cell apoptosis, we found that sno, positive regulator of Notch signaling pathway, was significantly up-regulated. However, the proteins involved in apoptotic pathway, such as CG7387, were significantly down-regulated. Furthermore, the protein level of CG17470, associated with mesoderm development [18], was also significantly decreased after *ocn* knockdown in fly testes (Fig. 2C). These indicate that some key signaling pathways involved in cell survival and differentiation are interfered in the *ocn* knockdown flies.

### Protein-protein interaction (PPI) analysis of DEPs

To further gain insight into the interactions of the DEPs, we constructed the protein-protein interaction (PPI) networks. Since *ocn* knockdown led to testes with no germ cells, we wondered whether these DEPs were involved in testis development. Therefore, we selected the top 50 DEPEs based on the network ranking score to construct PPI. The network contained proteins that were all down-regulated after the *ocn* knockdown in fly testes (Fig. 2D). Among them, many are associated with the processes of protein phosphorylation and dephosphorylation, such as CG4546, CG9389, and Ppi1. The 48 proteins in the inner circle revealed direct interactions with Ocn protein (Fig. 2D).

**Fig. 3:**
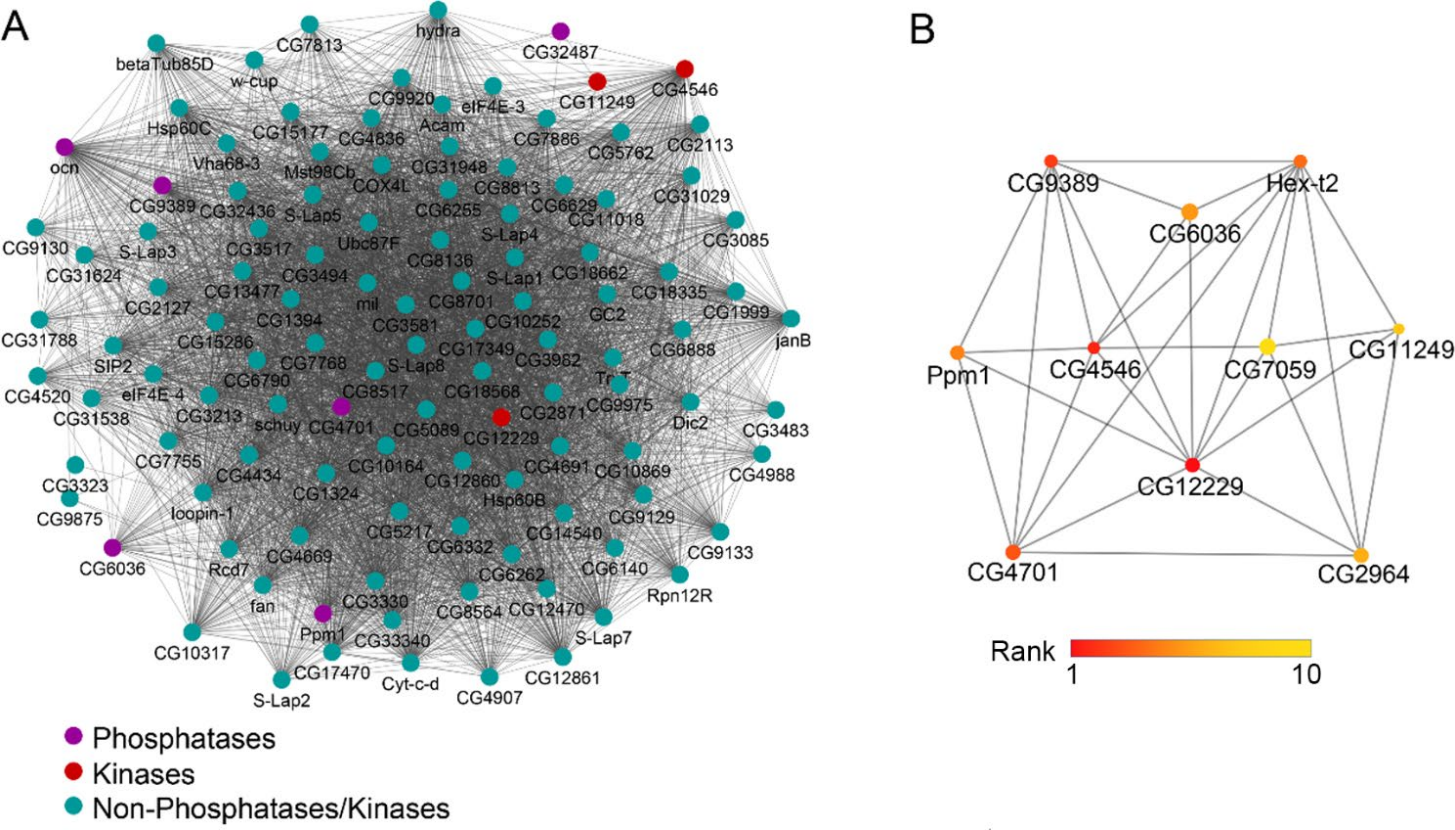
Protein-Protein interaction (PPI) between phosphorylation related enzymes (including16 phosphatases and 14 kinases) in DEPs and other DEPs. **(A)** PPI of Ocn interacting proteins. Purple indicates phosphatase, red indicates kinase, and green indicates other DEPs. **(B)** The top 10 proteins extracted from A based on the network ranking score obtained by MCC (maximum clique centrality). The color depth represents the rank of the score (importance) of the protein in the network: the darker the color (the darker red point), the higher the ranking (more important); the lighter the color (the lighter yellow point), the lower the ranking (less important)

Considering that Ocn is a phosphohistidine phosphatase, which might be involved in peptidyl-histidine dephosphorylation, we constructed an interaction network between phosphorylation related enzymes (including16 phosphatases and 14 kinases) among DEPs and other DEPs. We found 27 enzymes involved in phosphorylation could interact with 201 DEPs, forming 943 interactions (Fig. 3A). Ocn interacted with 3 kinases and 5 phosphatases. These 8 enzymes formed 687 interactions with 82.37% (187 / 227) DEPs in this network (Fig. 3A), suggesting an important role of phosphatases/kinases networks and interactions with Ocn. Based on the MCC (maximum clique centrality) network ranking scores in the phosphatase/kinase interacting network, the top ten proteins include seven proteins (CG12229 (kinase), CG4546 (kinase), CG9389 (kinase), CG4701 (phosphatase), CG6036 (phosphatase), Ppm1 (phosphatase), and CG11249 (kinase)) that interact with Ocn (Fig. 3B). Furthermore, according to single cell expression data from *Drosophila* testis [5, 19], the genes coding for these seven proteins are expressed in several stages of spermatogenesis, such as the GSC and early spermatogonia, late spermatogonia, and early spermatocytes, where *ocn* is also transcribed [5]. The functions of these seven genes in fly spermatogenesis have not been reported except for *CG4546*, which is essential for sperm development [20]. These results suggest that these kinases/phosphatases may play important roles in spermatogenesis through interaction with Ocn.

### Comparative analysis of proteomic and transcriptomic results

**Fig. 4:**
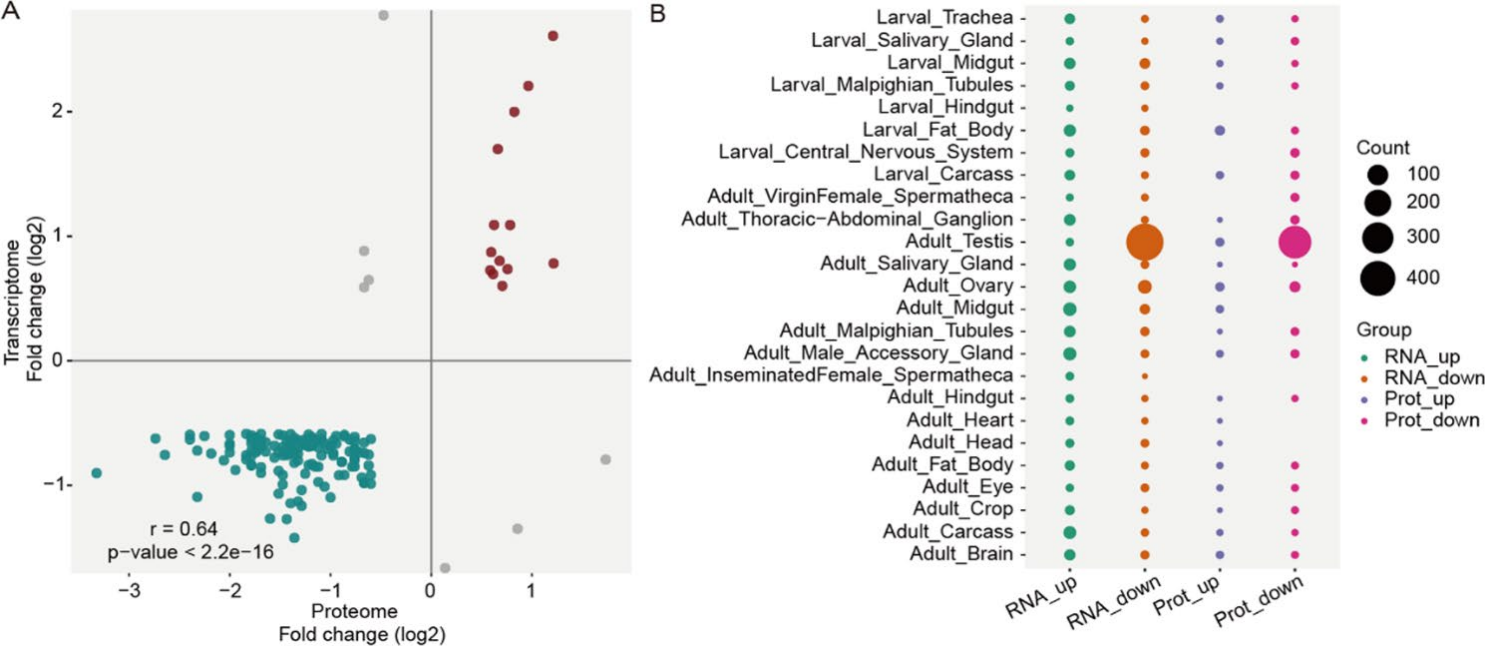
Comparison of DEPs and DEGs after *ocn* knockdown. **(A)** Correlation analysis between DEGs and DEPs after *ocn* knockdown. The correlation coefficient r = 0.64, *P* < 2.2E-16. **(B)** Many down-regulated DEGs and DEPs after *ocn* knockdown were testis-specific or highly expressed in the testis of *D. melanogaster*

To explore the consistency and relevance between proteome results and transcriptome results, we re-analyzed the transcriptome after *ocn* knockdown reported by our previous research [9] using DESeq2 with the criteria of a fold-change of > 1.5 and *P* < 0.05 (our previous analyses used a stricter criterion with a fold-change ≥ 2 and q-value < 5%). From 12,276 expressed genes (low-expressing genes are filtered), 1189 differentially expressed genes (DEGs) were identified. Of which, 373 genes were up-regulated and 816 genes were down-regulated after *ocn* knockdown. We found that 150 DEGs appeared in the DEPs identified in this study, and their changing trends in expressions after *ocn* knockdown were consistent (Fig. 4A). Furthermore, many common down-regulated DEGs and DEPs were testis-specific or highly expressed in the testis of *D. melanogaster* (Fig. 4B). These data suggest that *ocn* has a critical role in the development of testis.

### qRT-PCR validation

**Fig. 5:**
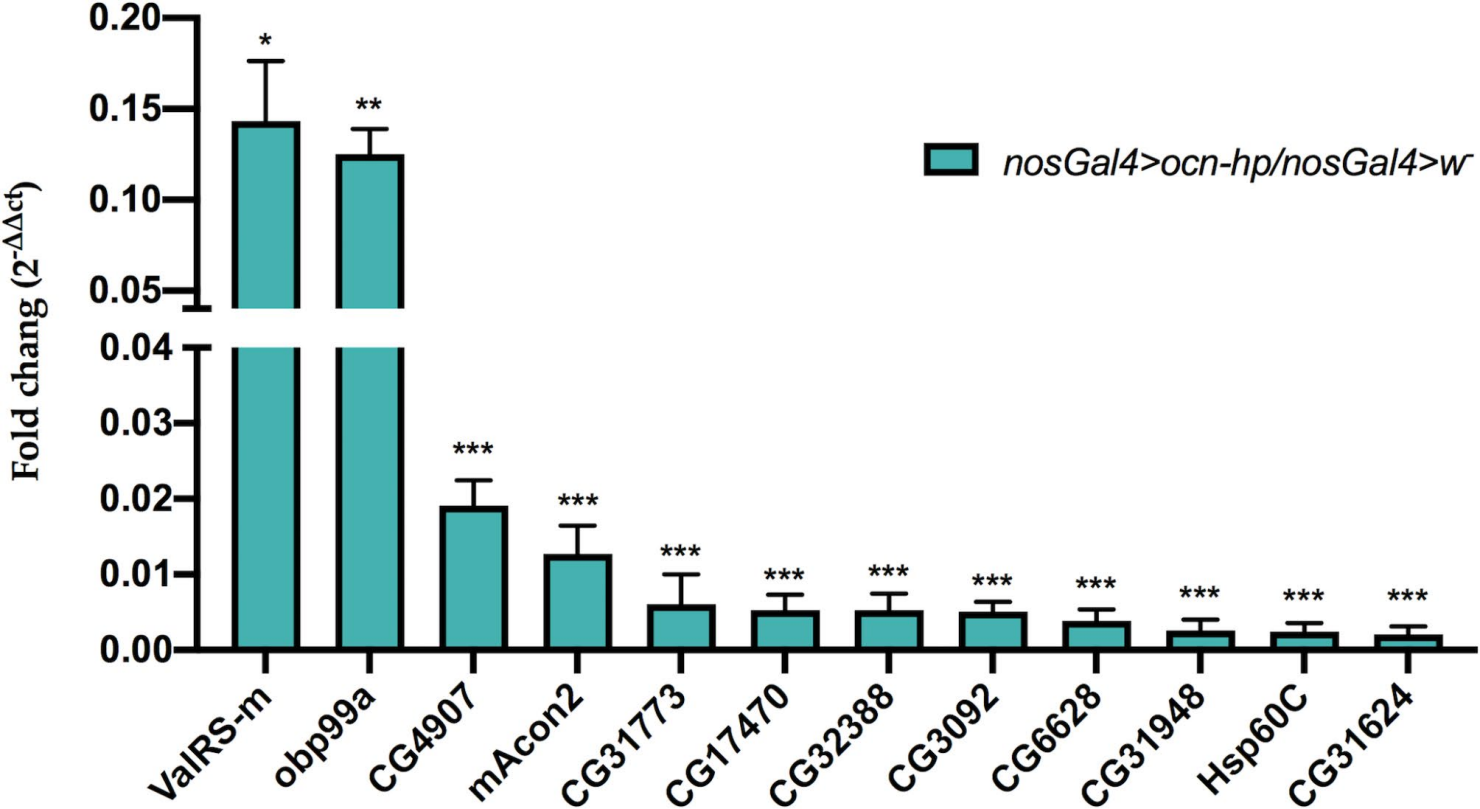
qRT-PCR validation of differentially expressed genes in testes selected from the DEGs and DEPs between *ocn* knockdown and control fly testes. “/” represents the relative value, bars indicate standard error. *, **, and *** indicate significant differences with *P* < 0.05, *P* < 0.01, and *P* < 0.001, respectively

To further verify the results of the proteomic analyses and to identify genes that may be involved in fly testis development, 12 genes, including *ValRS-m*, *obp99a*, *CG4907*, *mAcon2*, *CG31773*, *CG17470*, *CG32388*, *CG3092*, *CG6628*, *CG31948*, *Hsp60C*, and *CG31624* were selected for qRT-PCR to further investigate their expression profiles. The selection criteria for further expression analysis were based on their consistent appearance in both DEGs and DEPs in addition to specific or high expressions in fly testis (Flybase). The result showed all of the 12 genes were significantly down-regulated (*P* < 0.01 or *P* < 0.05) after *ocn* knockdown (Fig. 5). These changing trends are consistent with proteomic and transcriptomic results, indicating that knockdown of *ocn* in testes could cascade, directly or through its effects on testis development, into the decrease of the expression of a series of genes involved in testis development.

### Identification and quantification of phosphopeptides

**Fig. 6:**
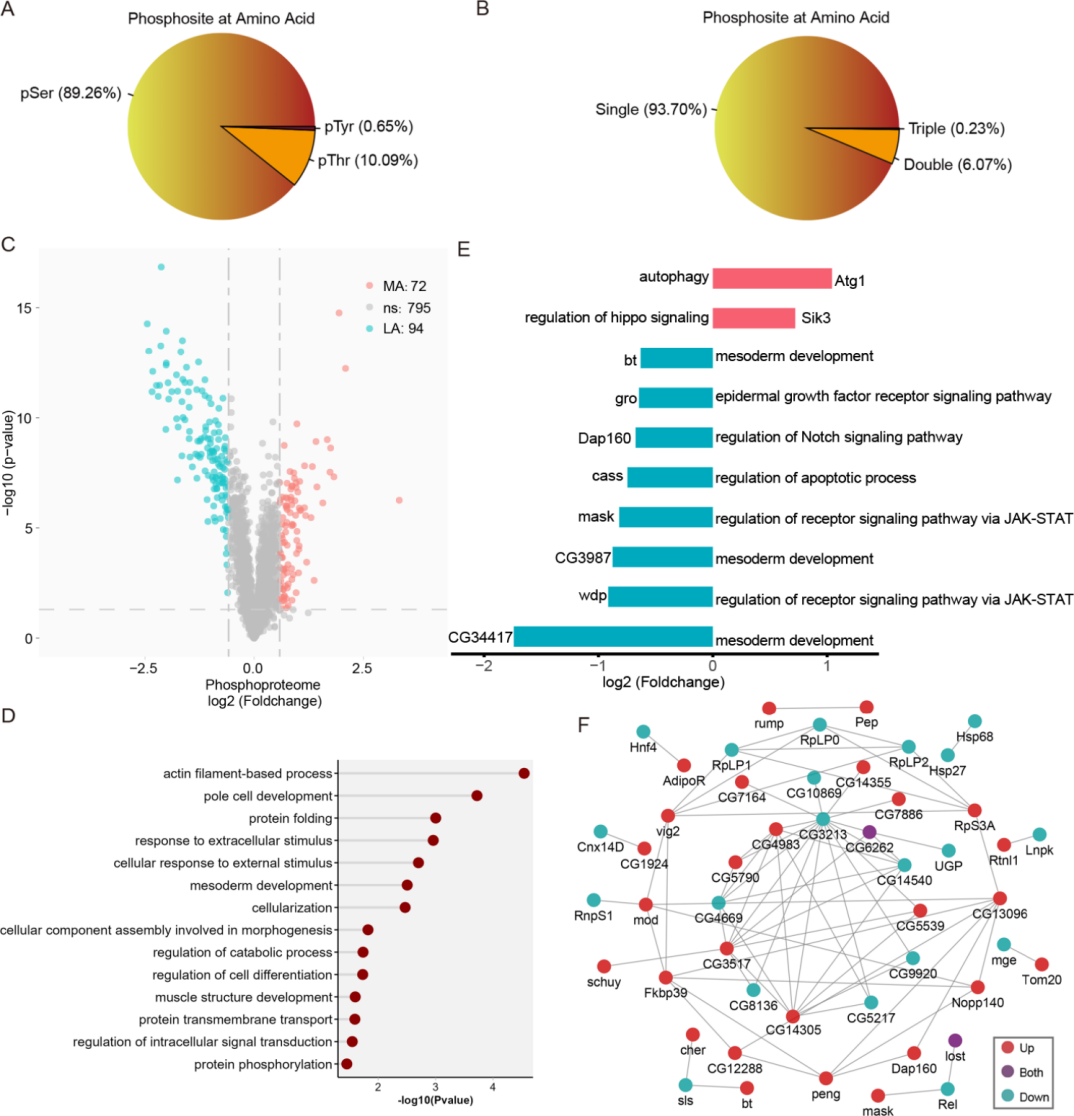
Analysis of differentially expressed phosphorylated proteins (DEPPs) between the *ocn* knockdown and control male abdomens of *D. melanogaster*. **(A)** Distribution of all quantified phosphorylated serine (pSer), threonine (pThr), and tyrosine (pTyr). **(B)** Distribution of phosphosite in phosphopeptides. **(C)** Volcano plot of differentially expressed phosphopeptides after *ocn* knockdown, cyan indicated down-regulation and red indicated up-regulation. **(D)** GO biological process enrichment analysis of DEPPEs (DEPPs after excluding spermatogenesis related proteins). **(E)** Log2 (fold change) of the expression levels of DEPPEs involved in key pathways in testis development. **(F)** Protein-protein interaction analysis of DEPPEs.

Considering that Ocn was predicted to have protein histidine phosphatase activity (flybase.org) and that protein phosphorylation is an important epigenetic modification which is critical for protein functions [21, 22], we compared the phosphoproteome of *ocn* knockdown and control male abdomens. We identified a total of 2,140 phosphorylated peptides (phosphors ≥ 0.75) located in 874 phosphoproteins with FDR < 1.0%. These 2,140 phosphorylated peptides included 89.26% of serine phosphorylation (pSer), 10.09% of threonine phosphorylation (pThr), and only 0.65% of tyrosine phosphorylation (pTyr) sites (Fig. 6A). Furthermore, among these phosphopeptides, most (93.70%) contained a single phosphorylated site, while the others carried multiple phosphorylation sites, including 6.07% with two sites (Fig. 6B).

Among the quantified proteins, a significant and at least 1.5-fold change were chosen as the cutoff for differential expressed phosphopeptides between the *ocn* knockdown and control male abdomens of *D. melanogaster*. Based on this criterion, 240 phosphorylated peptides, defining a total of 153 phosphoproteins were identified to be differentially expressed in the *ocn* knockdown fly abdomen. Among them, 102 phosphorylated peptides were up-regulated and 138 phosphorylated peptides were down-regulated (Fig. 6C, Additional file 2). Correspondingly, 72 phosphorylated proteins were up-regulated and 94 phosphorylated proteins were down-regulated. Thirteen phosphoproteins appeared in both up- and down-regulated groups, as they had multiple phosphorylation sites. These data indicate that differentially expressed phosphoproteins (DAPPs) may have changed in phosphorylation state, thus damaging their functions, and contributing to *Drosophila* testis developmental problems in the *ocn* knockdown flies.

To analyze the biological functions of the DEPPs, we performed GOBP enrichment analysis on them, and the results showed that the DEPPs were enriched in multiple testis development-related processes including male gamete production and myofibril assembly. We also analyzed the DEPPs after excluding spermatogenesis related proteins (DEPPEs) and found that the DEPPEs involved in actin filament-based process, protein folding, response to extracellular stimulus, and mesoderm development were significantly enriched (Fig. 6D). Again, to investigate the effect of *ocn* knockdown on testis development, we extracted DEPPEs in pathways involved in germ cell differentiation and tissue homeostasis, we found that the phosphorylation level of Atg1, which is the key regulator of autophagy pathway [23], was significantly up-regulated, while the phosphorylation levels of proteins, including bt, CG3987, and CG34417, associated with mesoderm development [18], were all significantly down-regulated. Furthermore, the phosphorylation levels of mask and wdp were also significantly decreased after *ocn* knockdown in fly testes (Fig. 6E). These two proteins are both involved in JAK-STAT pathway [24, 25]. These results suggest that some key signaling pathways involved in testis tissue homeostasis and germ cell differentiation are impacted in *ocn* knockdown flies.

To view the associations of the DEPPEs with testis development, based on the network ranking score we selected the top 50 DEPPEs to construct PPI. The network included 27 up-regulated and 21 down-regulated phosphoproteins after *ocn* knockdown. It also contained 2 phosphoproteins, CG6262 and lost, which appeared in both up- and down-regulated protein groups, since they had multiple phosphorylation sites (Fig. 6F).

**Table 1:** DEPs orthologs with human proteins involved in spermatogenesis

Dmel_uniprotID	Dmel_symbol	Human_symbol	GO
Q960D2	Cnb	CNTROB	GO:1,902,017, regulation of cilium assembly
Q24117	ctp	DYNLL2	GO:0097731, 9 + 0 non-motile cilium
Q7JVK6	trsn	TSN	GO:0001673, male germ cell nucleus
Q9VT31	CG16719	SPEF1	GO:1,990,716, axonemal central apparatus|GO:0097729, 9 + 2 motile cilium
Q8T417	TTLL4B	TTLL4	GO:0005929, cilium
Q06849	Arl2	ARL2	GO:0005929, cilium
Q9VKV8	Bug22	CFAP20	GO:0005879, axonemal microtubule
Q9VZH1	CG18675	CFAP298	GO:0005929, cilium
Q9VFH6	CG7886	CEP78	GO:0044782, cilium organization
Q9VV90	CG13032	CCDC13	GO:1,905,515, non-motile cilium assembly
Q8MSJ9	CG9313	DNAI1	GO:0005929, cilium
Q9W0M5	CG13901	DPCD	GO:0003351, epithelial cilium movement involved in extracellular fluid movement|GO:0007283, spermatogenesis
Q9W3J8	CG10958	DRC1	GO:0070286, axonemal dynein complex assembly
Q9VZZ8	CG16984	ENKUR	GO:0097728, 9 + 0 motile cilium|GO:0097228, sperm principal piece
Q9VS00	CG10064	CFAP52	GO:0030317, flagellated sperm motility
P41043	GstS1	HPGDS	GO:2,000,255, negative regulation of male germ cell proliferation
Q9W1V2	CG3085	TEKT2	GO:0030317, flagellated sperm motility
Q9VU41	Zmynd10	ZMYND10	GO:0003341, cilium movement
Q9VK29	Rsph1	RSPH1	GO:0007286, spermatid development|GO:0031514, motile cilium
Q9VA28	CG15547	NME5	GO:0007283, spermatogenesis|GO:0005929, cilium
Q9W1D3	Rsph4a	RSPH4A	GO:0003341, cilium movement
Q8T3Z0	Tektin-C	TEKT1	GO:0031514, motile cilium
Q8T476	Rsph3	RSPH3	GO:0005929, cilium
Q9VGG6	Dnali1	DNALI1	GO:0036126, sperm flagellum|GO:0031514, motile cilium
A1ZB91	Dnaaf3	DNAAF3	GO:0044458, motile cilium assembly
M9PCD3	CG8086	ODF3	GO:0007283, spermatogenesis
O76922	aub	PIWIL1	GO:0007283, spermatogenesis
Q9VGB6	Pglym87	PGAM2	GO:0007283, spermatogenesis
X2J6U8	ssp3	SCAPER	GO:0007283, spermatogenesis
Q9VH07	pont	RUVBL1	GO:0007283, spermatogenesis|GO:0120293, dynein axonemal particle
Q6GKZ1	klhl10	KLHL10	GO:0007286, spermatid development
A4V1Q1	bol	BOLL	GO:0007283, spermatogenesis
Q9V3K3	rept	RUVBL2	GO:0120293, dynein axonemal particle
Q9VH94	nmdyn-D7	NME7	GO:0005879, axonemal microtubule
Q9VBA1	Spag1	SPAG1	GO:0070286, axonemal dynein complex assembly
Q9VXP5	Efhc1.1	EFHC2	GO:0005879, axonemal microtubule
Q9VE97	CG7131	SAXO2	GO:0036126, sperm flagellum
Q7KVA7	Dhc62B	DNAH12	GO:0005858, axonemal dynein complex
Q9VIX9	CG17349	PACRG	GO:0097225, sperm midpiece|GO:0005879, axonemal microtubule
Q9VTP5	CG7264	RIBC2	GO:0005879, axonemal microtubule

### Motif analysis

**Fig. 7:**
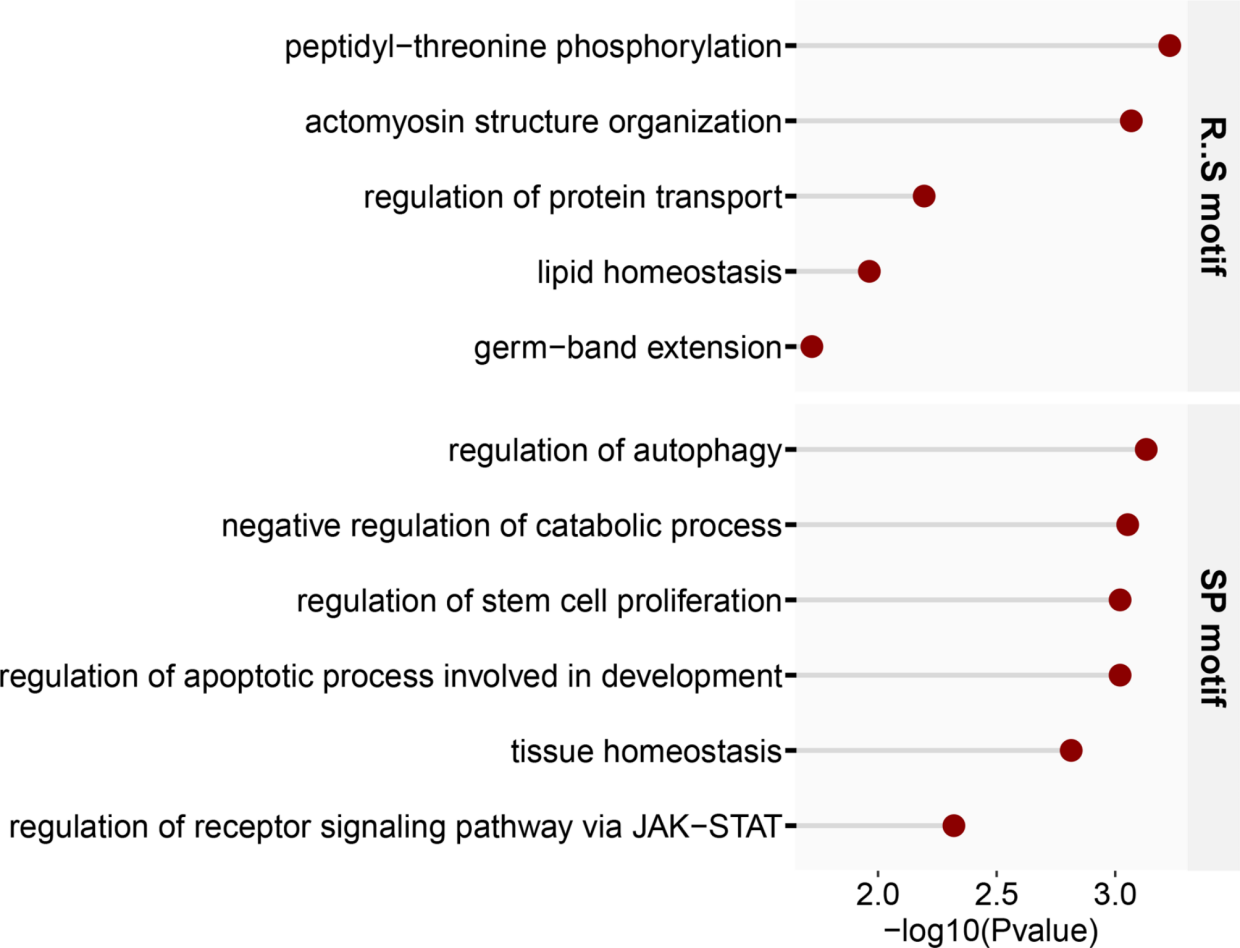
Motif analysis for DEPPs after excluding spermatogenesis related proteins (DAPPEs). Two motifs (RXXS and SP) were significantly enriched in DAPPEs. GO biological process enrichment analysis was performed for DAPPEs that significantly enriched in these two motifs, respectively (*P*adj < 0.05)

To gain insight into the potential functions of DEPPEs, we conducted motif analysis between − 7 and + 7 positions of phosphorylation sites and found that 2 motifs, RXXS and SP, were significantly enriched (*P*adj < 0.05) (Fig. 7). GOBP analyses revealed that those DEPPEs containing RXXS motif were involved in biological processes of peptidyl-threonine phosphorylation, actomyosin structure organization, regulation of protein transport, and lipid homeostasis. While those DEPPEs containing SP motif were associated with regulation of autophagy, negative regulation of catabolic process, regulation of stem cell proliferation, regulation of apoptotic process involved in development, and tissue homeostasis (Fig. 7). These suggest that the changes in protein phosphorylation states in *ocn* knockdown flies may play a crucial role in fly testis development *via* the phosphorylation-mediated signal pathway.

### Overlap between DEPs and DEPPs

**Fig. 8:**
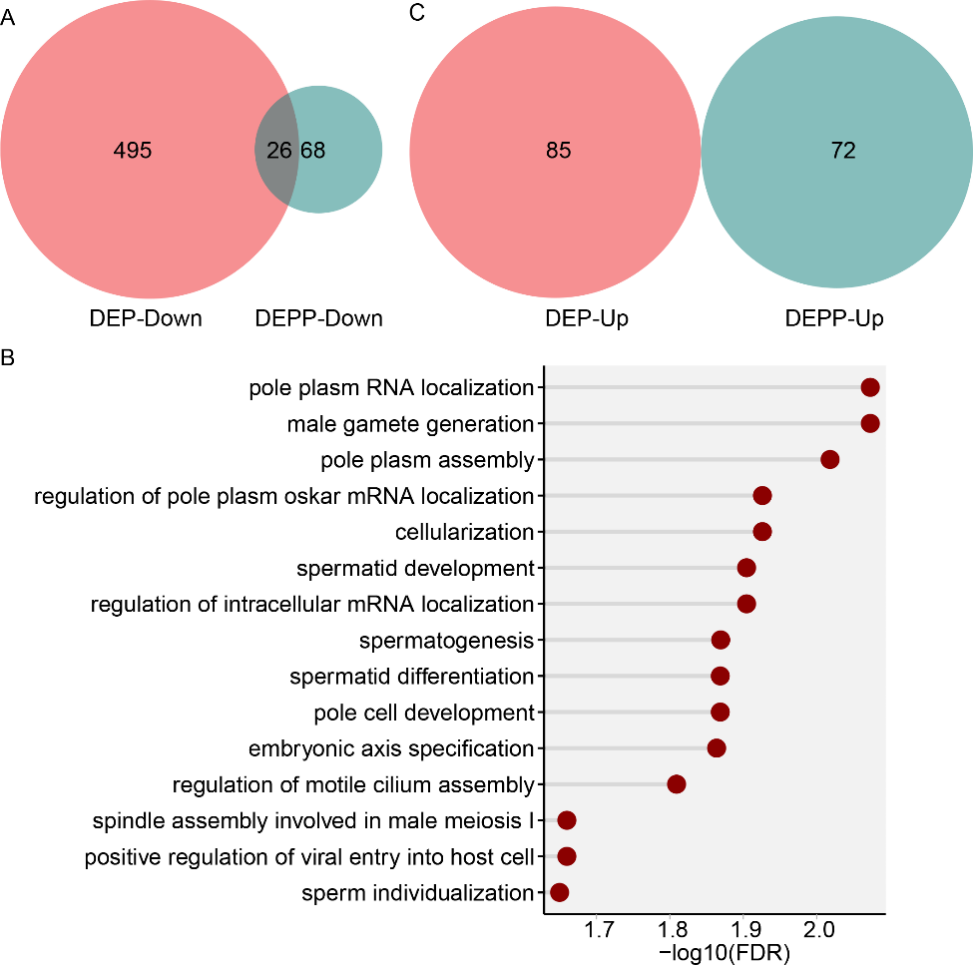
The correlation analysis between DEPs and DEPPs. **(A)** Venn diagram of the correlation numbers between down-regulated DEPs and down-regulated DEPPs. **(B)** GO biological process enrichment analysis of the 26 overlapped proteins between DEPs and DEPPs. **(C)** Venn diagram of the correlation numbers between up-regulated DEPs and up-regulated DEPPs.

We found very limited overlap between down and upregulated proteins between DEPs and DEPPs. Only 26 proteins were commonly downregulated from among 521 down-regulated DEPs and 94 down-regulated DEPPs (Fig. 8A). GOBP analyses for the commonly downregulated 26 proteins revealed that proteins involved in pole plasm RNA localization and male gamete generation were most significantly enriched (Fig. 8B), reflecting severe defects in male germ cell development. Noticeably, 68 out of a total 94 DEPPs (72.3%) were not even found among the mis-regulated DEPs. For example, Nup358 (Nucleoporin 358kD), an E3 SUMO-protein ligase, was significantly down-regulated (0.66-fold) in phosphorylation level, while there was no significant change in protein expression (0.99-fold) in the *ocn* knockdown fly abdomen. Moreover, 495 out of 521 (95.0%) DEPs were not downregulated in DEPPs. For instance, CG5270 was dramatically down-regulated in protein level (0.1-fold), but notably up-regulated (10-fold) in protein phosphorylation level by the *ocn* knockdown. None of the 85 and 72 up-regulated DEPs and DEPPs were commonly upregulated (Fig. 8C).

### Orthology with human proteomes

To investigate whether our data (DEPs and DEPPs) may provide potential candidates for further studies on their functions in male reproduction of animals, including humans, we searched for the human orthologs of the DEPs and DEPPs identified above in Flybase (FB2023_01, released February 15, 2023). This revealed that over 23% (140/606) of the DEPs has human orthologs. Based on GO annotations, 40 proteins showed involvement in male germ cell development (Table 1). Furthermore, over 39% (60/153) of the DEPPs has human orthologs. Based on GO annotations, 7 phosphoproteins displayed associations with male germ cell development (Table 2). These indicate that the DEPs and DEPPs identified in this research might also play important roles in testis development in other animals, including humans.

## Discussion

As a new gene specifically expressed in the testis of *D. melanogaster*, little was known about *ocn* function in testis development, although it was previously predicted that it may have histidine phosphatase catalytic activity (flybase.org). Our previous work has shown that knockdown of *ocn* driven by nosGal4 resulted in male sterility with extremely small testes containing no germ line but extended somatic hub cells in adult males [17]. The proteomics and phosphoproteomics strategies are powerful tools for the global analysis of functional networks in defined biological systems [26–28]. Therefore, in this study, through comparative proteomics and phosphoproteomics analyses, we identified 606 DEPs in *ocn* knockdown fly abdomens, of which 85 proteins were up-regulated and 521 were down-regulated after *ocn* knockdown. Moreover, 153 DEPPs were identified, with 72 up-regulated and 94 down-regulated phosphoproteins after *ocn* knockdown (13 phosphoproteins appeared in both up- and down-regulated groups owing to having multiple phosphosites). Many of these DEPs and DEPPs are likely involved in testis development.

Among the 521 downregulated DEPs, over 100 are sperm proteins found in *D. melanogaster* sperm proteome [7]. Furthermore, the re-analysis of the transcriptome also showed most genes as downregulated and predominantly expressed in the testis. These results, of both large number of transcripts and proteins downregulation, might explain our previous observation that *ocn* knockdown triggers, directly or indirectly, severe defects in germ cell development in the testis of *D. melanogaster* [17]. Interestingly, many of the down-regulated DEPEs are proteins involved in generation of energy and mitochondrial transport. Mitochondrial homeostasis and sufficiency of energy supply are essential for testis development and germ cell development. During testis development, mitochondria serve as energy factories and are crucial for testis development [29, 30]. Impaired mitochondrial dynamics in the testis of *D. melanogaster* impacts GSC maintenance through interfering with lipid homeostasis in a cell-autonomous manner [31]. Recently, Zhang et al. showed that knockout of mitochondrion-related phospholipase coding gene, *pld6*, resulted in severe defects in mitochondrial fusion, and in maintenance and differentiation of GSCs and progenitor cells in zebrafish, leading to male sterility with no sperms in the testis [32]. Therefore, the disruptions of mitochondrial homeostasis and consequent insufficient energy supply could contribute to the defect in testis development and the loss of germ cells observed after *ocn* knockdown in fly testes.

In most animals, the gonad develops from the mesoderm during embryogenesis [18, 33]. The gonad normally contains different types of somatic cells that regulate and nurture the germ cells during gamete production [34]. We found that the expression levels of some proteins or phosphorylated proteins related to mesoderm development, such as CG17470, bt, CG3987, and CG34417 [18], were significantly down-regulated (Figs. 2C and 6E). *CG17470*, highly expressed in the testis of *D. melanogaster* (flybase.org), was further verified to be significantly down-regulated by qRT-PCR analysis (Fig. 4). These suggest that the significantly down-regulation of some of these proteins associated with mesoderm development may also contribute to the defects in the testis development observed after *ocn* knockdown.

In *Drosophila* testes, the somatic gonadal precursors (SGPs) can develop to either quiescent hub cells at the tip of the testes or the cyst stem cells, which will differentiate to cyst cells enclosing and supporting germ cells during spermatogenesis [10]. Both Notch and Epidermal growth factor receptor (Egfr) signaling regulate cell proliferation and differentiation [35]. Kitadatea and Kobayashi demonstrated that in the male embryonic gonad, Notch was activated in almost all SGPs to specify hub fate, while Egfr signaling from the germline was activated in posterior SGPs to inhibit hub differentiation. Thus, hub is restricted at the anterior tip while CySCs are to the left areas of the embryonic gonad [36]. Given that the depletion of *ocn* resulted in a much smaller testis with no germ cells but extended somatic hub cells, we focused on the analyses of DEPEs involved in germ cell differentiation and cell apoptosis. We found that both Notch signaling pathway and Egfr pathways were significantly disturbed (Fig. 2C). The strawberry notch (Sno) protein was significantly up-regulated. *Sno* (*Sbno* in vertebrates) encodes a nuclear protein that positively regulates Notch signaling pathway [37]. In *Drosophila*, *sno* participates with Notch pathway in facilitating many common developmental pathways, including embryogenesis, eye development, wing development, exhibiting its crucial role in *Drosophila* development. Many phenotypes caused by *sno* mutation can be rescued by an extra copy of wild-type *Notch* [38]. In *C. elegans*, *let765*/*sno* plays a role in promoting RAS-dependent vulval development [39]. Knockout of the *Sbno1* in mouse caused the arrest of embryogenesis at the preimplantation stage with no blastocoel [40]. These studies indicate that Sbno/Sno functions in different developmental pathways. Egfr was significantly down-regulated after *ocn* knockdown in fly testes, probably due to loss of germ cells. As mentioned above, in *Drosophila* embryonic gonad, Egfr may inhibit the expression of *Notch*, thus restrict the hub at the anterior tip of the male gonad. Therefore, the up-regulation of Notch signaling and the down-regulation of Egfr signaling might contribute to the extended hub signals observed in *ocn* knockdown fly testes. Many proteins involved in apoptotic pathways were identified in DEPEs and DEPPEs, suggesting this pathway could be disturbed after *ocn* knockdown. However, changes of their expression levels did not exhibit a consistent trend, thus the influence of *ocn* knockdown on apoptotic pathway remains unclear.

Since the Ocn protein has a Janus domain, and the Janus kinase/signal transducer and activator of transcription (Jak/Stat) signaling pathway is necessary for regulating GSCs establishment and maintenance in fly testis [41, 42], we speculated that the loss of germ cells in *ocn* knockdown fly testes may relate to alterations of the JAK/STAT pathway. In *Drosophila*, when a ligand binds to the receptor Domeless, the associated JAK is activated [43]. The phosphorylated receptor/JAK complex then phosphorylates and dimerizes the transcription factor STAT92E, leading it into the nucleus and thus regulating target gene expressions [42]. All these steps in the pathway are strictly controlled, any disruptions in the pathway activity causes developmental and hematopoietic defects in flies and other animals, including mammals, as it is a highly conserved regulatory pathway in development. Here we found that several proteins among DEPPEs, such as mask (multiple ankyrin repeats single KH domain) and wdp (Windpipe), that are regulators of JAK/STAT signaling pathway [24, 25], were significantly changed in phosphorylation states. This indicates that in *ocn* knockdown *Drosophila* testes, JAK/STAT signaling pathway is hampered, thus affecting the stem cell establishment and maintenance and testis development.

Although caution should be exercised as spermatogenesis is one of the fastest evolving reproductive developmental processes, some proteins related to *Drosophila* spermatogenesis do have orthologs in mammals. By analysis of annotated *Drosophila* orthologs, Wasbrough et al. have indicated that over 65% of the updated *D. melanogaster* sperm proteome (DmSP-II) has mammalian orthologs including both mouse and human datasets [7]. In addition, several studies have demonstrated functional conservation of proteins involved in spermatogenesis. For example, *Drosophila* Cbc and its mammalian homolog CLP1 have been shown to be functionally conserved in subcellular localization and male fertility, mouse *CLP1* could even rescue the defects of male germ cell viability and male fertility caused by *cbc* mutation [44]. Here, we found that over 23% of the DEPs and over 39% of DEPPs had human orthologs. Some of them are involved in male germ cell development [44, 45]. Thus, our results provide a significant panel of candidates to investigate the underlying mechanisms of testis development in animals, including humans.

There is a possible limitation that the DEPs and DEPPs identified in this paper could be allometric results, since *ocn* knockdown fly testes showed much smaller size with no germ cells but extended hub cells. Nevertheless, these changed expressions of protein and phosphoproteins are trickle effects of the *ocn* knockdown. Thus, our results suggest that *ocn* is crucial for testis development and that its down-regulation ends up interfering key signaling pathways related to cell survival and differentiation.

## Conclusions

In this study, by comparative quantitative proteomic and phosphoproteomic assays we identified 606 differentially expressed proteins and 153 differentially expressed phosphoproteins in the abdomen of *D. melanogaster* after *ocn* knockdown in the testis. In addition to the DEPs associated with spermatogenesis, many other DEPs were involved in mitochondrion homeostasis, Notch signaling pathway, and apoptotic process, and showed interactions with Ocn. Furthermore, apart from the DEPPs that are related to spermatogenesis, many other DEPPs were involved in actin filament process, mesoderm development, autophagy, and JAK/STAT pathway. These suggest that *ocn* knockdown may trigger, directly or indirectly, the disruptions of energy supply, germ cell differentiation and/or maintenance, and thus testis development through ending up several important signaling pathways, including Notch, JAK/STAT, and cell apoptotic/autophagy pathways. Our data may provide significant information for understanding the molecular mechanisms underlying the male reproductive tissue development. These results could also contribute to the insights into animal testis development for future study.

## Methods

### Fly lines

Flies were reared on a standard cornmeal/yeast diet at 25 °C and under non-crowded conditions (200 ± 10 eggs per 50 ml vial of media in 150 ml conical flask) [46]. The *ocn* RNAi fly line (*ocn-hp*, Hairpin ID: TR04534P.1) was purchased from Tsinghua Fly Centre (Beijing, China), which interferes with the position from the base 373 to 393 of mRNA sequence. The *ocn* RNAi-2 fly line (*ocn-hp-2*, V12920) was purchased from Vienna Drosophila Resource Center (VDRC), which targets the region from 90 to 396 in mRNA sequence. The overexpressing line (*UAS-ocn*) was from FlyORF (F002764). The *nosGal4/Tm6B* flies were kindly provided by Professor Zhaohui Wang at the Institute of Genetics and Developmental Biology, Chinese Academy of Sciences. The rescue fly line (*ocn- hp-2*; *UAS-ocn*) with both *ocn* RNAi and overexpression elements was generated by a sequence of crossing. The detailed protocols were shown in Additional file 3. The hairpin construct in the RNAi lines or CDS construct in overexpressing line were expressed under the control of *nosGal4*. Transgenic *ocn-hp* or *UAS-ocn* male flies were crossed with virgin *nosGal4* females to obtain *ocn* knockdown flies (*nosGal4 > ocn-hp* and *nosGal4 > ocn-hp-2*) or overexpressing flies (*nosGal4 > UAS-ocn*). The male flies (*ocn-hp-2*; *UAS-ocn*) were crossed with *nosGal4* virgin females to obtain *ocn* rescue flies (*nosGal4 > ocn- hp-2*; *UAS-ocn*). Flies derived from the cross between *w*^*1118*^ males and *nosGal4* females (*nosGal4 > w*^*–*^) were used as controls.

### Fertility test

For each biological replicate, 1-day-old males (1d; n = 15) were left to mate with around 4d *w*^*1118*^ virgin females (n = 10) overnight (∼12 h). The following males were used in this experiment:

(1) *nosGal4 > w*^*−*^ (control), (2) *nosGal4 > ocn-hp-2* (knockdown), (3) *nosGal4*＞*cyo/+*; *UAS-ocn* (overexpression), and (4) *nosGal4*＞*ocn- hp-2*; *UAS-ocn* (rescue). Eggs were then collected and incubated at 25 °C and 45–70% humidity for around 30 h. Hatching rates were determined by counting the number of hatched eggs to total eggs. At least three biological replicates per cross type were performed.

### Protein extraction, iTRAQ labeling and titanium dioxide (TiO2) phosphopeptide enrichment

The protein extraction, iTRAQ labeling, TiO2 phosphopeptide enrichment, and LC-MS/MS were performed as per Yuan et al. [47] and Mao et al. [9] with some modifications. Briefly, for each sample the whole abdomens from about 250 one-day-old male flies were used (as the *ocn* knockdown fly testis was too small to be dissected). Protein extracts were obtained from three biological repeats. The protein of each biological sample (500 µg) was digested by trypsin (Promega) with the ratio of protein: trypsin = 40:1 overnight at 37 °C. After trypsin digestion, peptides were desalted with a Strata X C18 column (Phenomenex) and vacuum-dried according to the manufacturer’s protocol. For proteomic analysis, 100 µg of each protein sample was used for labeling. Each nosGal4 > *w*^*−*^ (control) sample was labeled with iTRAQ tags 118, 119, and 121, respectively. Each *nosGal4 > ocn-hp* sample was labeled with iTRAQ tags 113, 114, and 115, respectively.

**Fig. 9:**
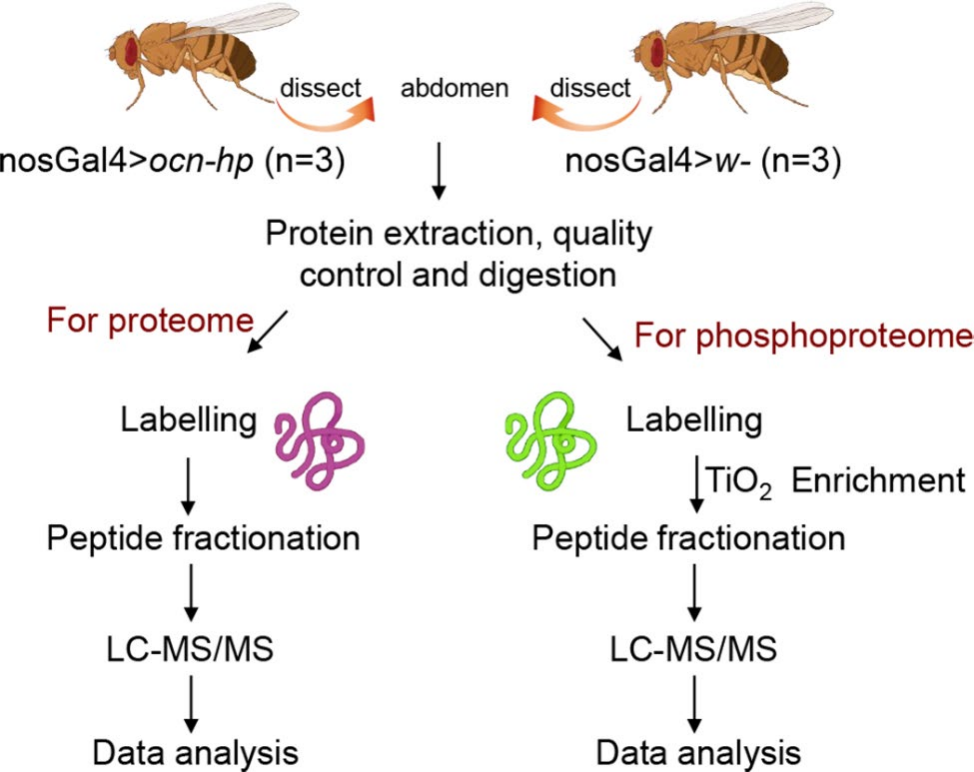
Experimental design and schematic diagram of the workflow. The abdomens from about 250 one-day-old strains of nosGal4 > *ocn*-hp and nosGal4 > *w*^*–*^ flies males was chosen for analysis of the differential proteomes. Then the same samples were analyzed by iTRAQ quantitative proteomics and phosphoproteomics, using the LC-MS/MS workflow. After thorough statistical analyses of the proteomics data, the differentially expressed proteins and phosphopeptides/sites were used for the subsequent bioinformatics analysis

For phosphoproteomic analysis, 400 µg of protein was used for labeling. The *nosGal4 > w*^*−*^ samples were labeled with iTRAQ tags 118, 119, and 121, while the *nosGal4 > ocn-hp* samples were labeled with iTRAQ tags 115, 116, and 117, respectively. The labeled peptides were desalted and concentrated using Sep-Pack C18 Cartridges (Waters, Milford, MA, USA). Then, 300 µg of iTRAQ-labeled peptides were added into freshly prepared TiO2 beads at peptides-to-beads ratio of 1:4 for phosphopeptide enrichment. The peptide-beads’ slurry was shaken on a rotator at 37 °C for 1 h and then centrifuged for 30 s and washed. Phosphopeptides were finally eluted with elution buffer for further analysis. The schematic diagrams of quantitative proteomics and phosphoproteomics analysis used in this study are shown in Fig. 9.

**Table 2:** DEPPs orthologs with human proteins involved in spermatogenesis

Dmel_uniprot	Dmel_symbol	Human_symbol	GO			
Q9VM92	TTLL3B	TTLL3	GO:0030317, flagellated sperm motility|GO:0036126, sperm flagellum			
X2J6U8	ssp3	SCAPER	GO:0061827, sperm head|GO:0007283, spermatogenesis|			
Q9VFH6	CG7886	CEP78	GO:0044782, cilium organization			
Q95RF6	Tom20	TOMM20	GO:0097225, sperm midpiece			
Q9VT31	CG16719	SPEF1	GO:1,904,158, axonemal central apparatus assembly| GO:0003341, cilium movement|			
Q6GKZ1	klhl10	KLHL10	GO:0007286, spermatid development|GO:0007286, spermatid development			
Q9W1D3	Rsph4a	RSPH4A	GO:0003341, cilium movement|GO:0060294, cilium movement involved in cell motility|GO:0031514, motile cilium			

### LC-MS/MS

The peptides were run on a Shimadzu’s LC-20AD model nano-liquid chromatograph. The extracted peptide samples were dissolved in mobile phase A (2% ACN, 0.1% FA), centrifuged at 20,000 g for 10 min. The supernatant samples were loaded onto a trap column for enrichment and desalination, and then were connected in series with a self-packed C18 column and separated by different gradients at a flow rate of 300 µl/min. Specifically, a 5% mobile phase B (98% ACN, 0.1% FA) for 0–8 min, a 8–21% mobile phase B for 8–76 min, a 21–32% mobile phase B for 76–82 min, a 32–80% mobile phase B for 82–85 min, a 80% mobile phase B for 85–90 min, and a 5% mobile phase B for 90–95 min. The peptides were ionized by the nanoESI source and then enter in the tandem mass spectrometer Q-Exactive (Thermo Fisher Scientific, San Jose, CA) for data-dependent acquisition (DDA) with following main parameter settings: ion source voltage 1.6 kV, the scanning range of the primary mass spectrum: 350 ~ 1600 m/z, and full scan resolution at 70,000, the initial m/z of the secondary mass spectrum 100 m/z, and MS/MS scans resolution at 17,500. The screening conditions for the precursor ions of the secondary fragmentation were: the charge 2 + to 7+, and the peak intensity of more than 10,000 ranked in the top 20 precursor ions. The ion fragmentation mode was HCD (high-energy collisional dissociation), and fragment ions were detected in Orbitrap. The dynamic rejection time was set to 15 s. Automatic gain control (AGC) target and maximum injection time were 1E5 and 40 ms, respectively. The datasets generated and analyzed during the current study are available in the ProteomeXchange Consortium (http://proteomecentral.proteomexchange.org) with the dataset identifier PXD036049.

### Data analyses

The raw MS/MS data were converted into MGF format by the corresponding tool, and the exported MGF files were searched by the local Mascot server (version 2.3) against the uniprot *D. melanogaster* 2018 protein database (41,166 sequence), including UniProtKB/ SWISS-PROT and UniProtKB/TrEMBL. Quality control (QC) was performed to determine if a reanalysis step was needed. An automated software IQuant was used for quantitatively analyzing the labeled peptides with isobaric tags [48], which combined a post processing tool of protein identification with advanced statistical algorithms for proteomics quantification filtered with 1% FDR (Benjamini and Hochberg-corrected *P* values) to obtain reliable phosphopeptides and phosphoproteins. Phosphorylation sites of the identified phospho-peptides were scored using phosphoRS provided within Proteome Discoverer (version 1.4), and filtered with 1% FDR (Benjamini and Hochberg-corrected *P* values) to obtain a reliable phosphorylation sites with phosphoRS probability ≥ 0.75. Proteins and phosphopeptides with at least a 1.5-fold change (mean value of all comparison groups) after quantification and *P*-value (t-test of all comparison groups) < 0.05 were defined as differentially expressed proteins (DEPs) and differentially expressed phosphoproteins (DEPPs).

### RNA preparation and quantitative reverse transcription PCR (qRT–PCR)

Total RNAs were extracted from 1-day-old (1d) nosGal4 > *ocn-hp* and nosGal4 > *w*^*−*^ adult testes using TRrizol reagent (Invitrogen®) according to the manufacturer’s recommendation. The first-strand cDNA was synthesized from 2 µg of total RNA samples using EasyScript One-Step gDNA Removal and cDNA Synthesis SuperMix (TransStart®) at 42 °C for 30 min. qPCR was performed using a Miniopticon system (BioRad, USA) with a Platinum SYBR Green qPCR SuperMix (TransStart®). The qPCR cycling program was as following: 95 °C for 5 min, followed by 40 cycles of 95 °C for 10 s, 55 ~ 60 °C (depending on different primers) for 20 s and 72 °C for 20 s, then a melting curve was constructed from 55 to 98 °C. The relative expression of the gene was calibrated against the reference gene *rp49* using the 2^−ΔΔCT^ calculation method: ΔΔCt = (Ct, _Target_ − Ct, _rp49_) *ocn*-kd − (Ct, _Target_ − Ct, _*rp49*_) con. The specific primers for tested genes were designed based on the sequences from the Flybase (flybase.org) database (Additional file 4).

### Bioinformatics analyses

For RNA-seq data re-mining, dataset was obtained from Zheng et al. [17]. Differential expression re-analysis was performed with DESeq2 (version 1.30.1). Transcripts Per Million (TPM) was used for data normalization and visualization in R software (version 4.2.0) (https://cran.r-project.org/). The fold changes from transcriptome and proteome were compared for their expression consistence. The tissue for the DEGs was extract from Gene Expression Omnibus (GSE7763) and visualized by R software.

For function annotation, we performed deeply analysis of differentially expressed proteins and phospho-proteins. Enrichment analysis of Gene Ontology (GO) was performed in WebGestalt (http://www.webgestalt.org/) [49] using Uniport Swiss-Prot (TrEMBL and Swiss-Prot sets) as background with Benjamini & Hochberg multiple test adjustment (*P* < 0.05). Pathway enrichment was analyzed based on Flybase (flybase.org). Then the analyzed results were visualized by R software. Venn diagrams were drawn by online software BioVenn (http://www.biovenn.nl/). For the DEP-kinase network, *Drosophila* kinase was downloaded from GLAD database (www.flyrnai.org/glad). DEPs-kinase/phosphotase interactions were obtained from STRING database (https://cn.string-db.org/). Kinase-kinase interactions were removed from the network for highlighting the DEPs-kinase/phosphotase interactions. The protein-protein interaction (PPI) network was constructed using cytoHubba. The network scoring values were obtained based on MCC (Maximal Clique Centrality) method. For the display option, we set “Display the shortest path”. MCC is an algorithm in cytoHubba, a Cytoscape plugin. CytoHubba can accurately screen out important nodes in the network, and the MCC algorithm is an accurate method for predicting important targets [50]. According the network scores, the top 50 proteins were visualized by Cytoscape (version 3.8.2) [51].

Motif analysis was performed as described previously [9], and function annotation was obtained from WebGestalt as described above. Briefly, the sequence was centered on each phosphorylation site and extended to 15 amino acids (± 7 residues) based on Flybase (flybase.org) and *Drosophila* Proteome using R script. The motif width was set to 15, significance was 1E-6. Motif-x was chosen as the algorithm to discover motifs in MoMo web server (version 5.1.1) [52]. Variable positions were marked by ‘‘x’’, for example, RxxSP. The motif of differentially abundant phosphoproteins was also extracted by MoMo web server integrated with motif-x [52]. As MoMo was running using shuffled foreground peptides as the background, we used an adjusted *P*-value (*P*adj < 0.05) that takes into account the multiple testing inherent in the search performed by motif-x for selecting significant residue/position pairs in the motif. After the motif extraction, the DEPPs containing those motifs were separately extracted for enrichment analysis as described above.

For retrieving human orthologs of the DEPs and DEPPs, the protein IDs of DEPs and DEPPs were input into Flybase and *Homo sapiens* (Human) was selected for orthologs searching. Then the biological process of GO terms of those identifiers was obtained by the Flybase Batch download.

### Statistical analysis and data availability

For qPCR experiments, we performed three technical replicates for each biological replicate. Data were expressed as means ± SEM (n = 3, 3 biological replicates). Shapiro–Wilk test was used to determine whether data were normally distributed. Student’s t-test for normally distributed data and Mann–Whitney U-test for normally distributed data were used to compare two groups. Statistical analyses and figures were performed and generated by R software. Significance levels are represented as: *P < 0.05, **P < 0.01, ***P < 0.001.

## Electronic supplementary material

Below is the link to the electronic supplementary material.

Supplementary Material 1

Supplementary Material 2

Supplementary Material 3

Supplementary Material 4

## Data Availability

The datasets generated and analyzed during the current study are available in ProteomeXchange Consortium (http://proteomecentral.proteomexchange.org) with the dataset identifier PXD036049. All other data are available in this text and Additional files.
