# Supplementary material for: Quantitative proteomics and phosphoproteomics reveal insights into mechanisms of ocnus function in Drosophila testis development
Source: BMC Genomics. 2023 May 26;24:283. doi: 10.1186/s12864-023-09386-2 (PMC10224340; doi:10.1186/s12864-023-09386-2)
Supplement: Supplementary file 3 — Supplementary Material 3 [file 12864_2023_9386_MOESM3_ESM.doc]

**
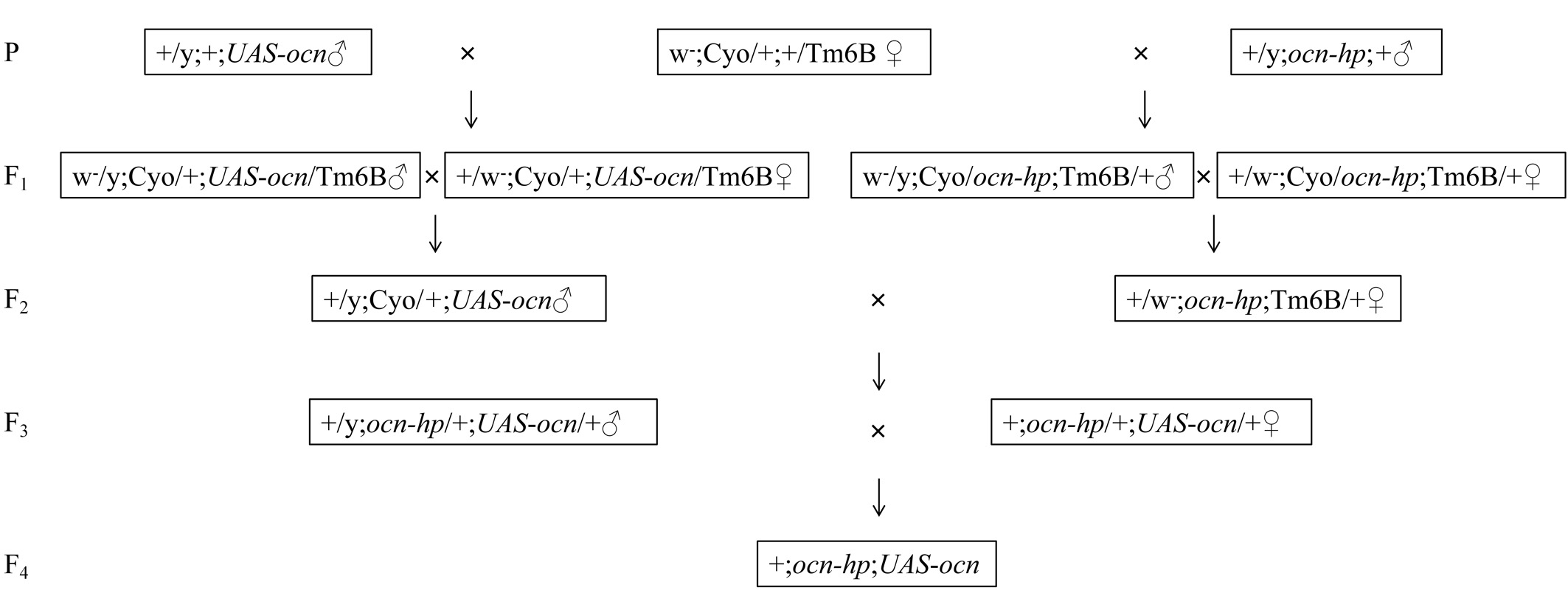
**

**Additional file 3** Schematic diagram of genetic hybridization used in this study to obtain the rescue line of *D. melanogaster*
