# Supplementary material for: Quantitative proteomics and phosphoproteomics reveal insights into mechanisms of ocnus function in Drosophila testis development
Source: BMC Genomics. 2023 May 26;24:283. doi: 10.1186/s12864-023-09386-2 (PMC10224340; doi:10.1186/s12864-023-09386-2)
Supplement: Supplementary file 4 — Supplementary Material 4 [file 12864_2023_9386_MOESM4_ESM.docx]

**Additional file 4** Primers used in this study

| Transcript | Forward primer (5ʹ–3ʹ) | Reverse primer (5ʹ–3ʹ) | |
| --- | --- | --- | --- |
| *ValRS-m* | CTAATTCAGCGGCGCAACTC | CGGCTCCGTGATCTCTACAC | |
| *Obp99a* | CGCCGACTATGTGGTGAAGA | CCACTTGGTGAAGACGCACT | |
| *CG4907* | CCAGCAGCAGAACAACCAAGG | AGGAGTATCAGTGCCGCATAG | |
| *mAcon2* | CCTTGGAACCGCTGGTGAATGG | TTGGCTATGCTGGCACATCGTC | |
| *CG31773* | CTGGGCGAAGGCGTCCATTATG | ATGTTGGCACACGGCGAAGTT |  |
| *CG17470* | TGAGTCGCCACCAGCAGAGAAT | TCGACGCCTACGACGGAACTAG |  |
| *CG32388* | GAAGGATTCGGGCAAGGATTCT | CGTGGTGAAGTGGGAACAACAG |  |
| *CG3092* | GAGGTGGTGGAGGTCAGTGAG | GCATCAGCCGTGTAGTCCCTTC |  |
| *CG6628* | GGATGTGCCGCCTTGAGATTCG | GGAACCGGACTGACAGCCAATG |  |
| *CG31948* | AGAACACCGCTGCCAGTCAATG | TGTCACCGAACTCCCACTCTCT |  |
| *Hsp60C* | TCAAGTCGGCACCTAGCATCG | TTAGGCGGTTCACCACCAGAGT |  |
| *CG31624* | AGAAGCCGCTCGCCAGTCAA | GCACCGCTGAGTCCGTTATAGG |  |
| *Rp49* | CGGTTACGGATCGAACAAGC | CTTGCGCTTCTTGGAGGAGA |  |
